# 4‐octyl itaconate alleviates cisplatin‐induced ferroptosis possibly via activating the NRF2/HO‐1 signalling pathway

**DOI:** 10.1111/jcmm.18207

**Published:** 2024-03-20

**Authors:** Li Zhang, Wenao Song, Hua Li, Xiaolin Cui, Jingyu Ma, Rongrong Wang, Yue Xu, Ming Li, Xiaohui Bai, Dawei Wang, Haihui Sun, Zhiming Lu

**Affiliations:** ^1^ Department of Laboratory Medicine Shandong Provincial Hospital Affiliated to Shandong First Medical University Jinan Shandong China; ^2^ Department of Laboratory Medicine, Shandong Provincial Hospital Shandong University Jinan Shandong China; ^3^ Department of Laboratory Medicine Weishan County Second People's Hospital Jining Shandong China; ^4^ Center for Reproductive Medicine, Cheeloo College of Medicine Shandong University Jinan Shandong China; ^5^ Department of Orthopaedics Shandong Provincial Hospital Affiliated to Shandong First Medical University Jinan Shandong China; ^6^ Department of Cardiology Shandong Provincial Hospital Affiliated to Shandong First Medical University Jinan Shandong China

**Keywords:** 4‐octyl itaconate, cisplatin, ferroptosis, NRF2, ototoxicity

## Abstract

Ferroptosis, characterized by iron‐dependent lipid reactive oxygen species (ROS) accumulation, plays a pivotal role in cisplatin‐induced ototoxicity. Existing research has suggested that in cisplatin‐mediated damage to auditory cells and hearing loss, ferroptosis is partially implicated. 4‐Octyl itaconate (4‐OI), derived from itaconic acid, effectively permeates cell membranes, showcasing potent anti‐inflammatory as well as antioxidant effects in several disease models. Our study aimed to investigate the effect of 4‐OI on cisplatin‐induced ferroptosis and the underlying molecular mechanisms. The survival rates of HEI‐OC1 cells and mice cochlea hair cells were measured by CCK8 and immunofluorescence, respectively. The auditory brainstem response (ABR) audiometry was used to detect changes in hearing thresholds in mice before and after treatment. Levels of ROS were evaluated by DCFH‐DA. Real‐time PCR quantified inflammatory cytokines TNF‐α, IL‐6 and IL‐1β. Network Pharmacology and RNA sequencing (RNA‐seq) analysis of the potential mechanism of 4‐OI resistance to cisplatin‐induced ferroptosis. The expressions of ferroptosis‐related factors (GPX4, SLC7A11 and PTGS2) and important antioxidant factors (NRF2, HO‐1, GCLC and NQO1) were tested by real‐time PCR, Western blot and immunofluorescence. Results demonstrated cisplatin‐induced significant ROS and inflammatory factor release, reduced NRF2 expression, hindered nuclear translocation and activated ferroptosis. Pretreatment with 4‐OI exhibited anti‐inflammatory and antioxidant effects, along with resistance to ferroptosis, ultimately mitigating cisplatin‐induced cell loss. In the present study, we show that 4‐OI inhibits cisplatin‐induced ferroptosis possibly through activation of the NRF2/HO‐1 signalling pathway, thereby exerting a protective effect against cisplatin‐induced damage to auditory cells, and providing a new therapeutic strategy for cisplatin‐induced hearing loss.

## 1 INTRODUCTION

Hearing loss has become one of the most prevalent sensory disorders in the world, which seriously affects the quality of life of patients.^1^ Increasing ageing problems, excessive noise exposure and long‐term use of ototoxic drugs are common causes of hearing loss. Cisplatin is a common type of antitumor drug in ototoxic drugs, which disrupts the DNA synthesis function of cells to eventually kill cancer cells and has been used in the clinical treatment of many cancers.^2^, ^3^, ^4^ However, cisplatin has been shown to have serious toxic effects during clinical practice, including bilateral, progressive and irreversible hearing loss.^5^, ^6^, ^7^ Unfortunately, there is still no effective treatment available for this type of hearing loss that occurs in adults, while sodium thiosulfate is approved by the FDA in the United States for only use at the paediatric level.^8^, ^9^, ^10^ Previous studies considered apoptosis as the primary cause of cisplatin‐mediated cellular ototoxicity.^11^, ^12^, ^13^ Recent investigations, however, have revealed that ferroptosis occurs during the cisplatin‐induced ototoxicity.^14^, ^15^

Ferroptosis is a novel type of cell death that differs from other forms of cell death, for example, apoptosis, necrosis and autophagy,^16^, ^17^ which is characterized by the presence of intracellular accumulation of iron ions and the generation of a large amount of reactive oxygen species (ROS), accompanied by lipid peroxidation, morphological changes and function loss of mitochondria.^18^, ^19^ Some rescue strategies for ferroptosis have been explored. For example, ferrostatin‐1, a specific inhibitor of ferroptosis, has been shown to prevent cisplatin‐induced cell loss.^20^, ^21^ Nuclear factor erythroid 2‐related factor 2 (NRF2) is a transcription factor of an important antioxidant defence system in the body.^22^ Under normal circumstances, NRF2 binds to KEAP1 and is anchored in the cytoplasm. Under oxidative stress in the body, NRF2 is released to enter the nucleus to activate downstream target gene transcription in the nucleus, such as genes encoding heme oxygenase‐1 (HO‐1) and glutathione peroxidase 4 (GPX4).^22^, ^23^ Previous research has demonstrated that oxidative stress was strongly associated with cisplatin‐induced ototoxicity, with NRF2 acting as a key element to prevent oxidative stress.^24^ Both in vivo and in vitro studies have shown that cisplatin‐induced ototoxicity was attenuated through the NRF2 signalling pathway.^25^ The researchers found that cisplatin‐induced ferroptosis could be attenuated by activating the NRF2 signalling pathway in a model of cisplatin‐induced acute kidney injury.^26^ Furthermore, studies of other diseases have confirmed that the activation of the NRF2/HO‐1 signalling pathway could enhance the ability to resist oxidative stress in the body and improve GPX4 levels, thereby inhibiting cell ferroptosis.^27^, ^28^ Therefore, we propose that inhibition of ferroptosis through activation of the NRF2 signalling pathway may ameliorate cisplatin‐induced hearing loss.

As an NRF2 specific activator, 4‐octyl itaconate (4‐OI) is a derivative of itaconic acid.^29^ As a type of endogenous metabolite, the itaconic acid is responsible for the synthesis of aconitate decarboxylase encoded by immune‐responsive gene 1 (*Irg1*), which converts cis‐aconitate, an intermediate metabolite of the tricarboxylic acid (TCA) cycle, into itaconic acid, thereby involving in regulating the cellular immune metabolism.^29^, ^30^, ^31^ Furthermore, previous studies have demonstrated the ability of itaconic acid to significantly reduce the inflammatory response and oxidative stress.^32^ 4‐OI has been revealed to be protective in many disease models due to its high solubility and ease of passage through cell membranes compared to itaconate.^33^, ^34^, ^35^ For example, it has been reported that 4‐octyl itaconate caused a significant reduction in hepatic ischemia–reperfusion injury by activating NRF2‐mediated antioxidant responses in hepatocytes against inflammation and oxidative stress.^36^

Although 4‐OI has shown better therapeutic effects in many disease models, there have been no reports on the effect of 4‐OI on cisplatin‐induced ototoxicity as yet. Therefore, our goals of this study were (1) to explore the relationship between 4‐OI and cisplatin‐induced ototoxicity in vitro and in vivo using an animal model of cisplatin injury, the HEI‐OC1 cell line, and mouse cochlear explants, and (2) to determine the potential therapeutic effect of 4‐OI on cisplatin‐induced hearing loss.

## 2 MATERIALS AND METHODS

### 2.1 Cell culture and drug therapy

HEI‐OC1 cells were cultured in DMEM (Gibco, USA) medium containing 10% FBS under room temperature (about 33°C) and 10% CO_2_ to reach the density of 70%–90% and then to perform the passage experiments. In the experimental group, 4‐OI (250 μM; HY‐112675, MedChemExpress, USA) was added 3 h in advance. The cisplatin treatment was given at a concentration of 30 μM for 24 h. The Ras‐selective lethal 3 (RSL3) (HY‐100218A, MedChemExpress, USA) treatment was given at a concentration of 4 μM for 24 h.

### 2.2 Anatomy and culture of cochlear explants

The cochlea of C57BL/6J mice of 3‐ or 4‐day‐old were dissected in precooled phosphate buffer saline (PBS). The dissected basement membrane was attached to a glass coverslip coated with cell‐Tak adhesive (354240, BD Biosciences, USA), incubated in DMEM/F12 (1056018, Thermofisher, USA) mixed with N‐2 (17502048, Thermofisher, USA), B‐27 (17504044, Thermofisher, US) and ampicillin (A1170, Solarbio, Beijing). The samples were dosed after overnight incubation. In the experimental group, 4‐OI (300 μM) was added 6 h in advance. The cisplatin treatment was given at a concentration of 30 μM for 24 h. The study was reviewed and approved by the Animal Management and Ethics Review Committee of Shandong Provincial Hospital, China (Permit #2022–025).

### 2.3 Immunofluorescence staining of the HEI‐OC1 cells and mice cochlear explants

After the experimental treatments of each group, the HEI‐OC1 cells and cochlear basement membranes were fixed with 4% paraformaldehyde (PFA) for 30 min and were punched with 0.1% Triton X‐100 and 1% Triton X‐100 for 30 min, respectively. Then, the samples were treated with PBT‐1 blocking solution for 1 h, and incubated with PBT‐1 diluted primary antibody in a refrigerator (4°C) overnight. The primary antibody dilution was aspirated off and the samples were then incubated with PBT‐2 diluted secondary antibody and DAPI at 37°C for 1 h. The sample was sealed, observed and imaged under a fluorescent inverted microscope (Leica Microsystems, Biberach, Germany). The antibodies used included Myosin 7a (1:500, 138–1, DSHB, USA), NRF2 (1:200, A1244, ABclonal, China), HO‐1 (1:200, A1346, ABclonal, China) and GPX4 (1:200, AB125066, Abcam, UK).

### 2.4 Cell activity assay

Cell viability was determined using the CCK8 assay kit (CK04, Dojindo, Japan). The 96‐well plates were inoculated with HEI‐OC1 cell suspension with a cell density of 5000/well. Each group was treated with different doses of 4‐OI and cisplatin based on the experimental requirements. A total of 10 μL of CCK8 solution was added to each well, and incubated for 1 h at 37°C in the incubator. The OD value of the cells at 450 nm was measured and the cell viability was calculated.

### 2.5 ROS assay

Quantification of ROS in HEI‐OC1 cells and cochlear basement membrane was performed using the DCFH‐DA kit (S0033S, Beyotime Biotechnology, China). Diluted DCFH‐DA solution was added to each group and incubated for 30 min at 37°C. The nuclei were labelled with Hoechst, and the samples were observed and recorded with a fluorescent inverted microscope (Leica Microsystems, Biberach, Germany).

### 2.6 RNA extraction and real‐time PCR

The total RNA of each treatment group was extracted using AG RNAex Pro RNA Extraction Reagent (AG21101, Accurate Biotechnology, China) and reverse transcribed into cDNA. The real‐time PCR experiment was performed using SYBR® Green Pro Taq HS premixed qPCR Kit (AG11701, Accurate Biotechnology, China) with the primers provided in Table S1. GAPDH was used as an internal reference and the $2^{-\text{ΔΔCt}}$ method was used to calculate the gene expression levels.

### 2.7 Total cell protein extraction and Western blot

Each experimental group was washed twice with precooled PBS at 4°C, and the whole proteins were extracted by adding lysis solution RIPA (P0013, Beyotim, China) containing both phosphatase and protease inhibitors, lysed for 30 min at 4°C, vortexed every 5 min, and then centrifuged at 12,000*g* for 20 min at 4°C. The protein contents were measured with the BCA kit (P0010, Beyotime, China), added to the loading buffer, and then denatured by heating. After the protein samples were electrophoresed by 10% and 12% SDS‐PAGE, the proteins were transferred to PVDF membranes by wet transfer method. After blocking for 1 h, primary antibodies, including NRF2 (A1244, ABclonal, China), HO‐1 (A1346, ABclonal, China), NQO1 (A19586, ABclonal, China), GPX4 (ab125066, Abcam, UK), SLC7A11 (ab175186, Abcam, UK) and β‐actin (GB11001, Servicebio, China), all diluted 1:1000, were added and incubated overnight at 4°C. Then, the corresponding secondary antibody dilutions (1:5000) were added and incubated for 1 h. The target protein was analysed semiquantitatively using a fully automated chemiluminescence image analyser and ImageJ software for grayscale analysis of protein bands, with β‐actin as an internal reference.

### 2.8 Network pharmacology

The file in SDF format and SMILES of 4‐OI were obtained from the PubChem database (https://pubchem.ncbi.nlm.nih.gov/; accessed on June 30, 2023). The SuperPred and SwissTargetPrediction databases were utilized for 4‐OI target prediction.^37^, ^38^ The genes involved in ferroptosis induction and inhibition were obtained from the FerrDb V2 database.^39^ Venny diagram was performed using TBtools,^40^ while the protein–protein interaction (PPI) network was constructed using the STRING database and visualized using Cytoscape (v3.9.0).^41^, ^42^ Additionally, the MCODE plugin was employed to capture the subnetworks of PPI. Protein structures were retrieved from the PDB database (https://www.rcsb.org/; accessed on June 30, 2023), and molecular docking was conducted using AutoDock Vina 1.1.2. The interaction patterns of the docking results were analysed using PyMOL 2.3.0. Enrichment analyses based on both Gene Ontology (GO) and Kyoto Encyclopedia of Genes and Genomes (KEGG) databases were performed using the R package clusterProfiler (v3.14.3).

### 2.9 RNA‐seq analysis

A total of 1 × 10^6^ HEI‐OC1 cells were inoculated in 6‐cm culture plates and divided into three experimental treatment groups: the control, the cisplatin group and the 4‐OI (250 μM) + cisplatin group. The control cells were incubated at 33°C in a 10% CO_2_ incubator for 24 h, the cisplatin‐treated group was treated with 30 μM cisplatin for 24 h, and the 4‐OI + cisplatin group was treated with 250 μM 4‐OI for 3 h and then 30 μM cisplatin was added for a total of 24 h. The total RNA of each treatment group was extracted using AG RNAex Pro RNA Extraction Reagent (AG21101, Accurate Biotechnology, China). Both RNA purity and concentration were detected by NanoDrop 2000 spectrophotometer, with the RNA integrity precisely detected by Agient2100/LabChip GX. After the samples passed the quality test, cDNA library construction was carried out, and after the libraries passed the quality control, PE150 mode sequencing was performed using the Illumina NovaSeq6000 sequencing platform. Genes were screened using Fold Change ≥2 and FDR <0.01 as differential gene screening criteria to detect the differentially expressed genes (DEGs). Volcano map and heat map were generated using the online tool (https://hiplot.com.cn/). GO and KEGG metabolic pathway enrichment analyses were performed using standard computational enrichment methods. The sequencing data used in this study were submitted to the (NCBI; https://www.ncbi.nlm.nih.gov/sra/) and with the accession number PRJNA990464.

### 2.10 Extraction of cytoplasmic and nuclear proteins

Proteins in both cytoplasm and nucleus of HEI‐OC1 cells were extracted using the kits, respectively (R0050, Solarbio, China). First, HEI‐OC1 cells were collected by cell scraping, transferred into a precooled centrifuge tube, and centrifuged at 500*g* for 2–3 min. The supernatant was collected and the precipitate was kept for further use. The proper amount of plasma protein extraction reagent was added to disperse the cell precipitate into a single‐cell suspension. The supernatant was kept in an ice bath for 10 min and then centrifuged at 12,000–16,000*g* at 4°C for 10 min. The precipitate obtained contained the nuclei. An appropriate amount of nuclear protein extraction reagent was added to make the cell precipitate completely dispersed. After 10 min of the ice bath and centrifugation at 12,000–16,000*g* at 4°C for 10 min, the supernatant obtained contained the nuclear proteins.

### 2.11 Animals and drug treatments

The 8‐week‐old male C57BL/6J mice were procured from Jiangsu Huachuan Pharmaceuticals Co., Ltd. The 24 mice were randomly assigned to one of four groups: the control group (*n* = 6), the cisplatin group (*n* = 6), the 4‐OI + cisplatin group (*n* = 6) and the 4‐OI group (*n* = 6). These animals were housed in a standard laboratory environment maintained under Specific Pathogen‐Free (SPF) conditions. The animal facility was regulated with a 12‐h light–dark cycle, maintaining a relative humidity between 40% and 50%, and a stable temperature of 25 ± 1°C. Continuous access to both feed and water was provided, and the bedding was replaced daily. Throughout the experiment, researchers observed and recorded the daily activities, mental state and body weight of the mice. The intraperitoneal dosages of 4‐OI and cisplatin were in line with previous studies.^43^, ^44^ A 3 mg/kg stock solution of cisplatin was prepared in sterile saline (0.9% NaCl) at room temperature. 4‐OI was dissolved in (2‐hydroxypropyl)‐β‐cyclodextrin with PBS to create a 25 mg/kg solution of 4‐OI. The cisplatin group received a consistent dose of 3 mg/kg through intraperitoneal injections over 7 consecutive days. In the 4‐OI + cisplatin group, mice were administered 25 mg/kg of 4‐OI through intraperitoneal injection 12 h prior to cisplatin on the 1st and 4th days. The 4‐OI group received 25 mg/kg of 4‐OI through intraperitoneal injection on the 1st and 4th days. In addition, all mice undergoing cisplatin treatment received subcutaneous injections of 1 mL of physiological saline every 12 h throughout the experiment to prevent dehydration and renal toxicity. The control group received intraperitoneal injections of an equivalent volume of physiological saline for 7 consecutive days. The study was reviewed and approved by the Animal Management and Ethics Review Committee of Shandong Provincial Hospital, China (Permit #2022‐025).

### 2.12 Measurement of auditory brainstem response (ABR)

On both 1st day prior to the injection and the 10th day after the injection, ABR tests were conducted using TDT System III (Tucker‐Davis Technologies, Alachua, FL, USA). The anaesthetised mice were positioned within a soundproof chamber, with the recording electrodes delicately placed at the cranial vertex, reference electrodes at the postauricular mastoid, and grounding electrodes along the midline of their back. Testing was systematically carried out at frequencies of 4, 8, 12, 16, 24 and 32 kHz. The intensity levels varied from 90 dB to 10 dB, gradually decreasing in 10 dB increments, with the hearing thresholds recorded at each frequency. Subsequent to the tests, the mice were carefully situated on a 37°C heating pad and monitored until they fully regained consciousness.

### 2.13 Immunofluorescence staining of the cochlear basement membranes in mice

After conducting ABR tests on mice, euthanasia was performed under pentobarbital sodium anaesthesia. The head of each mouse was swiftly removed using surgical scissors, and the cochlea was collected and fixed in 4% PFA at room temperature for 1 h, then rinsed with PBS three times. Subsequently, the cochlea was immersed in 10% EDTA for decalcification for 48 h. Under a microscope, the basement membranes were dissected into apical, middle and basal turns. The dissected basement membrane was attached to a glass coverslip coated with cell‐Tak adhesive. The cochlear basement membranes were punched with 1% Triton X‐100 for 30 min. Then, the samples were treated with PBT‐1 blocking solution for 1 h and incubated with PBT‐1 diluted Myosin 7a in a refrigerator (4°C) overnight. The primary antibody dilution was aspirated off and the samples were then incubated with PBT‐2 diluted secondary antibody at 37°C for 1 h. The sample was sealed, observed and imaged under a fluorescent inverted microscope.

### 2.14 Data analyses

For each experimental group, three or more biological replicates were repeated and statistical analysis was performed using GraphPad Prism 8 software. Comparisons between groups were made using unpaired *t*‐tests and one‐way ANOVA. Analysis of the grayscale values and fluorescence intensity of the molecular bands were calculated using ImageJ software with the significant difference determined based on *p* < 0.05.

## 3 RESULTS

### 3.1 4‐OI reduces the toxic effect of cisplatin on HEI‐OC1 cells

**FIGURE 1:**
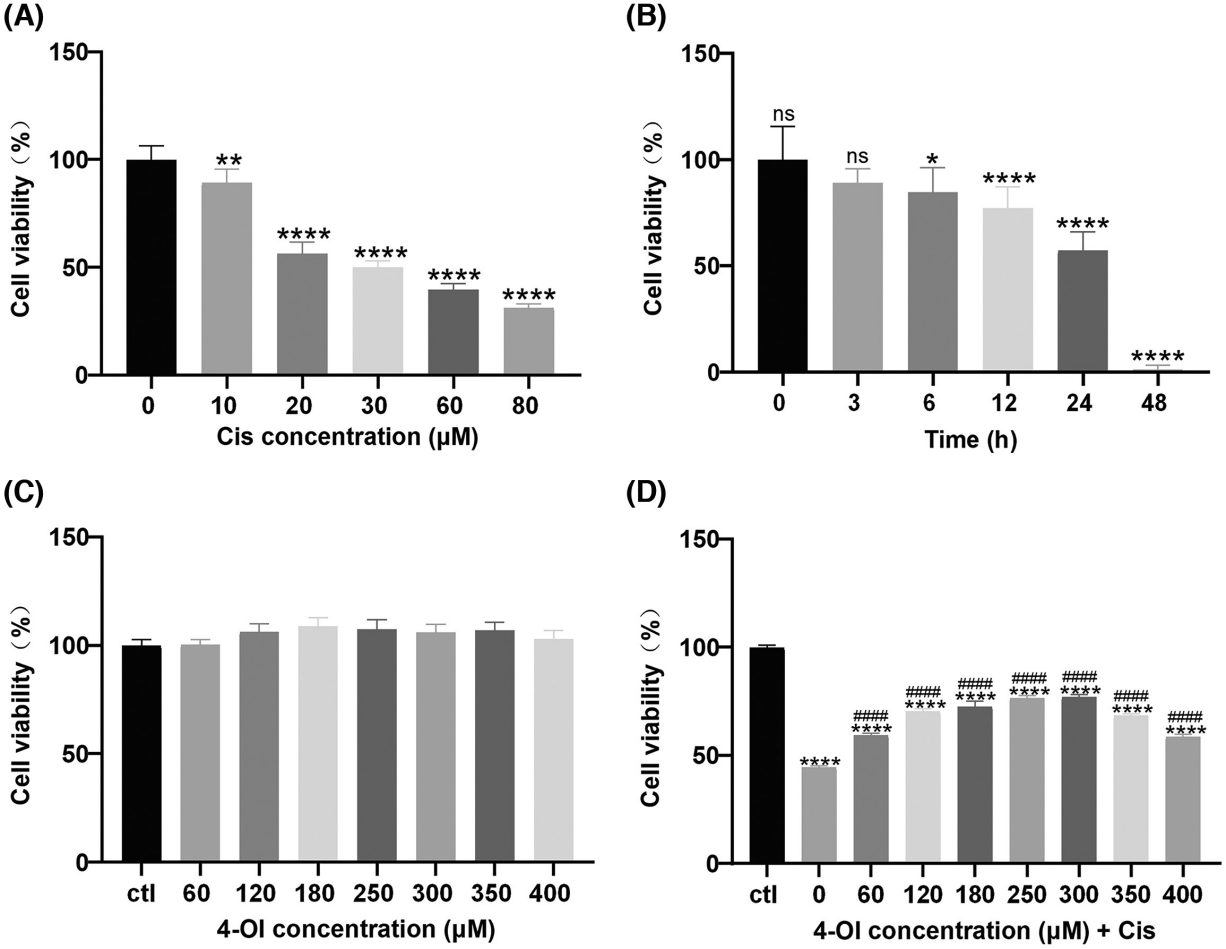
4‐OI alleviates the cisplatin‐induced decrease in HEI‐OC1 cell viability. (A) Cell survival rate by CCK8 assay after 24 h treatment of different concentrations of cisplatin (0, 10, 20, 30, 60 and 80 μM) on HEI‐OC1 cells. (B) CCK8 assay of cell survival after treatment of 30 μM cisplatin applied to HEI‐OC1 cells for different treatment times (0, 3, 6, 12, 24 and 48 h). (C) Cell survival rate detected by CCK8 assay after 24 h of 4‐OI treatment at different concentrations on HEI‐OC1. (D) Cell survival rate based on CCK8 of HEI‐OC1 cells first treated with different concentrations of 4‐OI for 3 h and then by 30 μM cisplatin for 24 h. The values are expressed as mean ± SEM (*n* = 3). The significant differences are determined by *p < 0.05, **p < 0.01 and ****p < 0.0001 versus control group, respectively, and by ^####^ *p* < 0.0001 versus cisplatin group. ‘ns’ indicates no significance; ctl, control; Cis, cisplatin.

The results of the CCK‐8 assay demonstrated a significant reduction in the survival rate of HEI‐OC1 cells to 50% following the 24 h treatment with 30 μM cisplatin (*p* < 0.05; Figure 1A,B). Therefore, a concentration of 30 μM cisplatin for a 24 h treatment duration was selected for subsequent experiments. Evaluation of the cytotoxicity of 4‐OI on HEI‐OC1 cells revealed no significant changes in cell survival rates within the range of 0–400 μM 4‐OI compared to the control group (Figure 1C), indicating the absence of cytotoxic effects at these doses of 4‐OI. Prior to the treatment of cisplatin, HEI‐OC1 cells were preincubated with various concentrations of 4‐OI (i.e. 0, 60, 120, 180, 250, 300, 350 and 400 μM) for 3 h, followed by incubation with 30 μM cisplatin for 24 h. The results revealed a strong protective effect of 4‐OI against cisplatin‐induced damage in HEI‐OC1 cells (Figure 1D). However, this protective effect diminished when the concentration of 4‐OI exceeded 300 μM. The highest cell viability in HEI‐OC1 cells was observed after the pretreatment of 4‐OI at 250 and 300 μM, with no significant difference in cell survival between these two concentrations. Therefore, a concentration of 250 μM 4‐OI was selected as the optimal protective level of 4‐OI for the subsequent cell experiments.

### 3.2 4‐OI reduces the toxicity of cisplatin on cochlear explants

**FIGURE 2:**
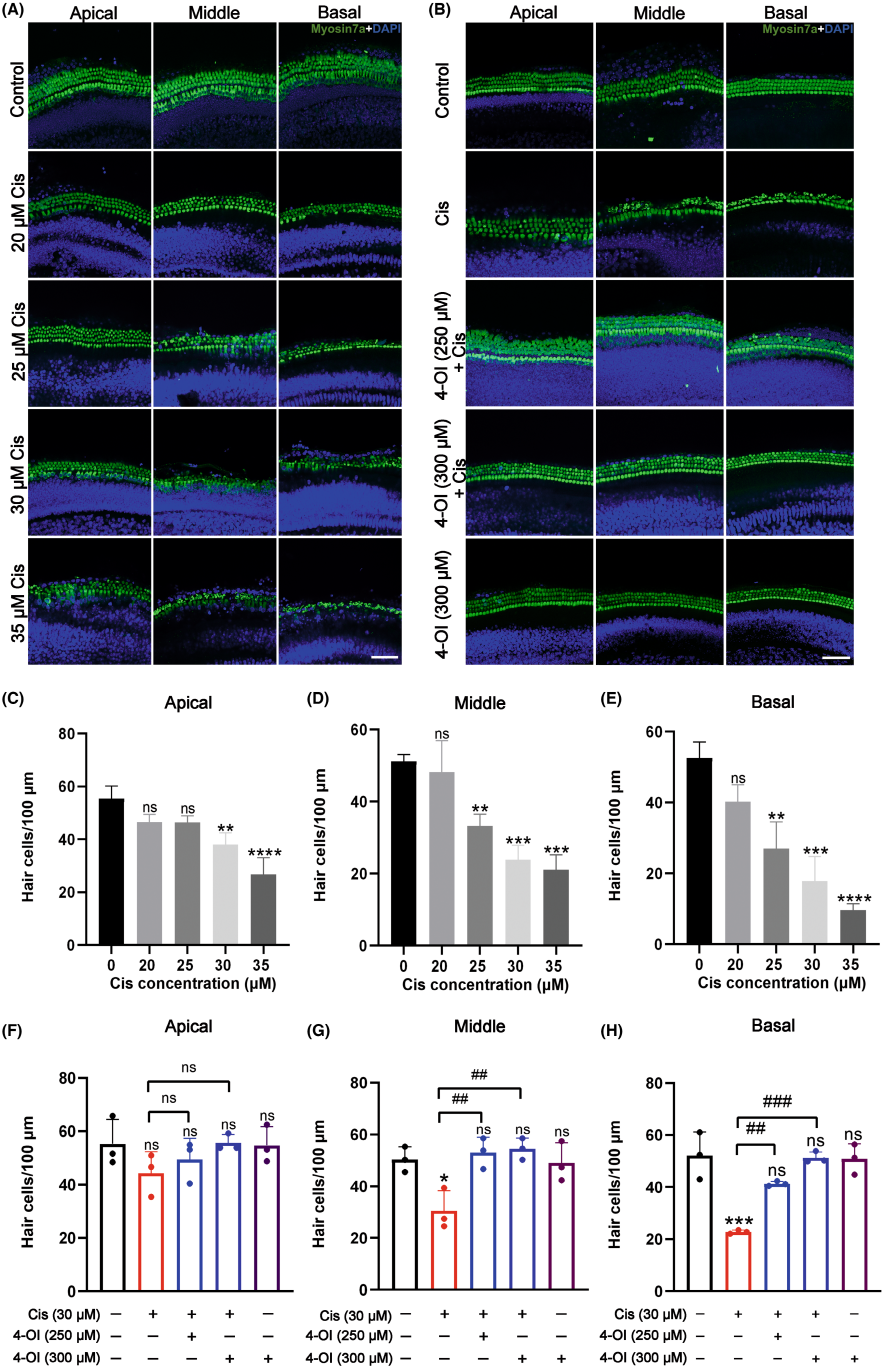
4‐OI mitigates cisplatin‐induced reduction in cochlear hair cell survival. (A, B) Representative images of Myosin 7a (green) and DAPI (blue) in the apical, middle and basal turns of the cochlear basement membrane with immunofluorescence staining in five groups of samples. (C–E) Number of Myosin 7a positive hair cells per 100 μm at the apical, middle and basal turns of the cochlear basement membrane in the five groups of samples shown in (A). (F–H) Number of Myosin 7a positive hair cells per 100 μm at the apical, middle and basal turns of the cochlear basement membrane in the five groups of samples shown in (B). The values are expressed as mean ± SEM (*n* = 3). The significant differences are determined by *p < 0.05, **p < 0.01, ***p < 0.001 and ****p < 0.0001 versus control group, respectively, and by ^##^ *p* < 0.01 and ^###^ *p* < 0.001 versus cisplatin group, respectively. ‘ns’ marks no significance. Scale bar = 40 μm. Cis, cisplatin.

To assess the protective effect of 4‐OI on cochlear explants, the optimal concentration of cisplatin to induce damage in cochlear explants was first determined. Different concentrations of cisplatin (i.e. 0, 20, 25, 30 and 35 μM) were applied to the basement membranes of mice for 24 h (Figure 2A). The confocal images labelled with Myosin 7a revealed a notable loss of hair cells in response to cisplatin treatment compared to controls. This loss occurred initially in the basal turn and progressively spread to the middle and apical turns, exhibiting a clear dose‐dependent pattern. Based on the total count of hair cells, the survival rate of apical hair cells exhibited a statistically significant decrease, while the survival rates of middle and basal turn hair cells were significantly dropped to 50% (*p* < 0.05; Figure 2C–E). Therefore, a concentration of 30 μM cisplatin was selected for subsequent experiments involving the basement membrane hair cells. The protective effects of 4‐OI on cisplatin‐induced ototoxicity were then assessed in five groups of samples: the control group, the cisplatin group, the 4‐OI (250 μM) + cisplatin group, the 4‐OI (300 μM) + cisplatin group and the 4‐OI (300 μM) group. The results showed that the pretreatment with 4‐OI for 6 h prior to cisplatin treatment significantly reduced the cisplatin‐induced cochlear hair cells loss (Figure 2B). Analysis of basement membrane hair cells count revealed no significant numerical or morphological differences in cochlear hair cells between the 4‐OI treatment and control groups, suggesting the absence of toxic effects of 4‐OI on cochlear explants. Moreover, the total number of surviving hair cells in the 4‐OI (300 μM) + cisplatin group was notably higher than that in the 4‐OI (250 μM) + cisplatin and cisplatin groups (*p* < 0.05; Figure 2F–H). Consequently, a concentration of 300 μM 4‐OI was chosen as the optimal treatment concentration for subsequent experiments involving cochlear hair cells.

### 3.3 4‐OI mitigates the cisplatin‐induced elevation of ROS and inflammatory factors

**FIGURE 3:**
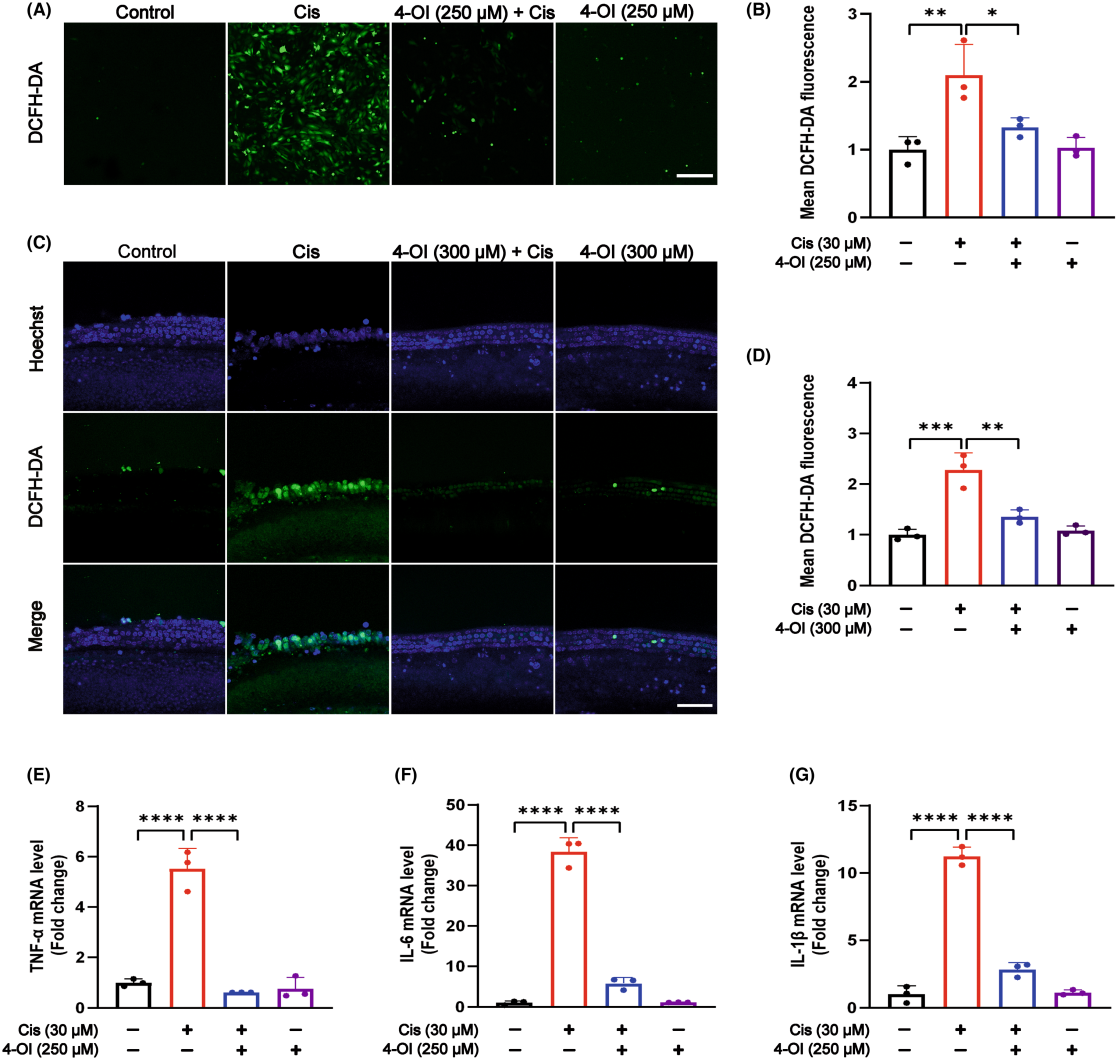
4‐OI reduces cisplatin‐induced ROS and inflammatory factors production and accumulation. (A) Four groups of samples stained for DCFH‐DA in live cells with DCFH‐DA used as a measure of intracellular ROS. (B) Quantitative analysis of DCFH‐DA immunofluorescence intensity of HEI‐OC1 living cells shown in (A). (C) Representative images of four groups of samples of the cochlear basement membrane transferred to DCFH‐DA (green) and Hoechst (blue) staining. (D) Quantitative analysis of DCFH‐DA immunofluorescence intensity in cochlear basement membrane shown in (C). (E–G) Relative mRNA levels of *TNF‐α*, *IL‐6* and *IL‐1β* in four groups of HEI‐OC1 cells. The values are expressed as mean ± SEM (*n* = 3). The significant differences are determined by *p < 0.05, **p < 0.01, ***p < 0.001 and ****p < 0.0001, respectively. Scale bar = 40 μm. Cis, cisplatin.

The results of DCFH‐DA fluorescence staining revealed a significant increase in green fluorescence signals in HEI‐OC1 cells of the cisplatin group compared to the control group, indicating excessive intracellular ROS production induced by cisplatin. Conversely, the 4‐OI pretreatment group exhibited a significant reduction in green fluorescence signals (Figure 3A,B). Immunofluorescence staining of cochlear hair cells with DCFH‐DA also showed an evident enhancement of green fluorescence signals in the cisplatin group compared to the control group, whereas the 4‐OI pretreatment group displayed a sharp decrease in green fluorescence signals (Figure 3C,D). The real‐time PCR analysis demonstrated the anti‐inflammatory effect of 4‐OI, as the mRNA expression of pro‐inflammatory cytokines, including *TNF‐α*, *IL‐6* and *IL‐1β*, was significantly enhanced in the cisplatin group compared to the control group. However, pretreatment with 4‐OI significantly reduced the expression of these pro‐inflammatory cytokines (*p* < 0.05; Figure 3E–G).

### 3.4 Cisplatin inhibits the NRF2 signalling pathway and activated ferroptosis in HEI‐OC1 cells

**FIGURE 4:**
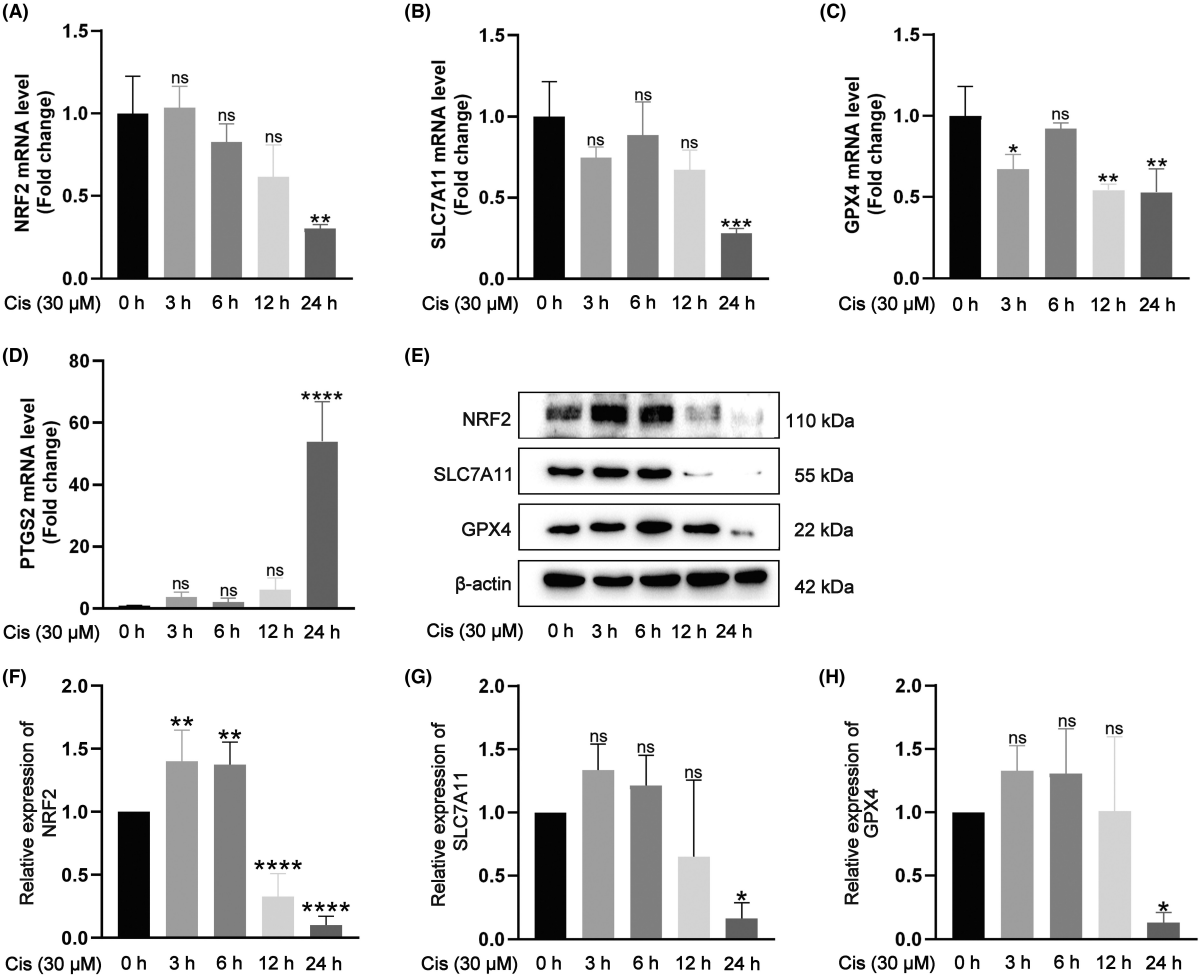
Cisplatin inhibits the NRF2 signal pathway and induces ferroptosis in vitro. (A–D) Relative mRNA levels of *NRF2*, *SLC7A11*, *GPX4* and *PTGS2* in five groups of HEI‐OC1 cells treated with cisplatin for 0, 3, 6, 12 and 24 h, respectively. (E) Protein expressions of NRF2, SLC7A11 and GPX4 in five groups of HEI‐OC1 cells. (F–H) Quantitative statistics of NRF2, SLC7A11 and GPX4 proteins shown in (E). The values are expressed as mean ± SEM (*n* = 3). The significant differences are determined by *p < 0.05, **p < 0.01, ***p < 0.001 and ****p < 0.0001 versus control group, respectively. ‘ns’ indicates no significance. Cis, cisplatin.

In order to further investigate the possible causes of ototoxicity induced by cisplatin, cisplatin of 30 μM was used to treat HEI‐OC1 cells for 0, 3, 6, 12 and 24 h, respectively, to assess the expression of the important antioxidant component NRF2 and the associated ferroptosis marker protein. The results of real‐time PCR and Western blot analyses showed that the expression of NRF2 was gradually declined as the duration of cisplatin treatment was increased. In 24 h, the expression of NRF2 was significantly lower than that of the control (*p* < 0.05; Figure 4A,E,F). Based on these results, we hypothesized that the NRF2 signalling pathway might play a regulatory role in the cisplatin‐induced cell damage. To further evaluate the relationships between cisplatin‐induced cell damage and ferroptosis, HEI‐OC1 cells were treated with 30 μM cisplatin for 0, 3, 6, 12 and 24 h, respectively. The results showed that the expressions of two key markers of ferroptosis, that is, GPX4 and solute carrier protein 7 family member 11 (SLC7A11) at both mRNA and protein levels, were continuously decreased with the extension of cisplatin treatment time (*p* < 0.05; Figure 4B,C,E,G,H). Moreover, prostaglandin‐endoperoxide synthase 2 (PTGS2), a potential marker of ferroptosis, showed a dramatic increase at mRNA level in 24 h after the cisplatin treatment compared to the control (*p* < 0.05; Figure 4D). These results clearly show that cisplatin elicits the injury of HEI‐OC1 cells mainly via triggering the pathway of ferroptosis.

### 3.5 Network pharmacology and RNA‐seq reveal a potential protective effect of 4‐OI on the cisplatin‐induced ferroptosis

**FIGURE 5:**
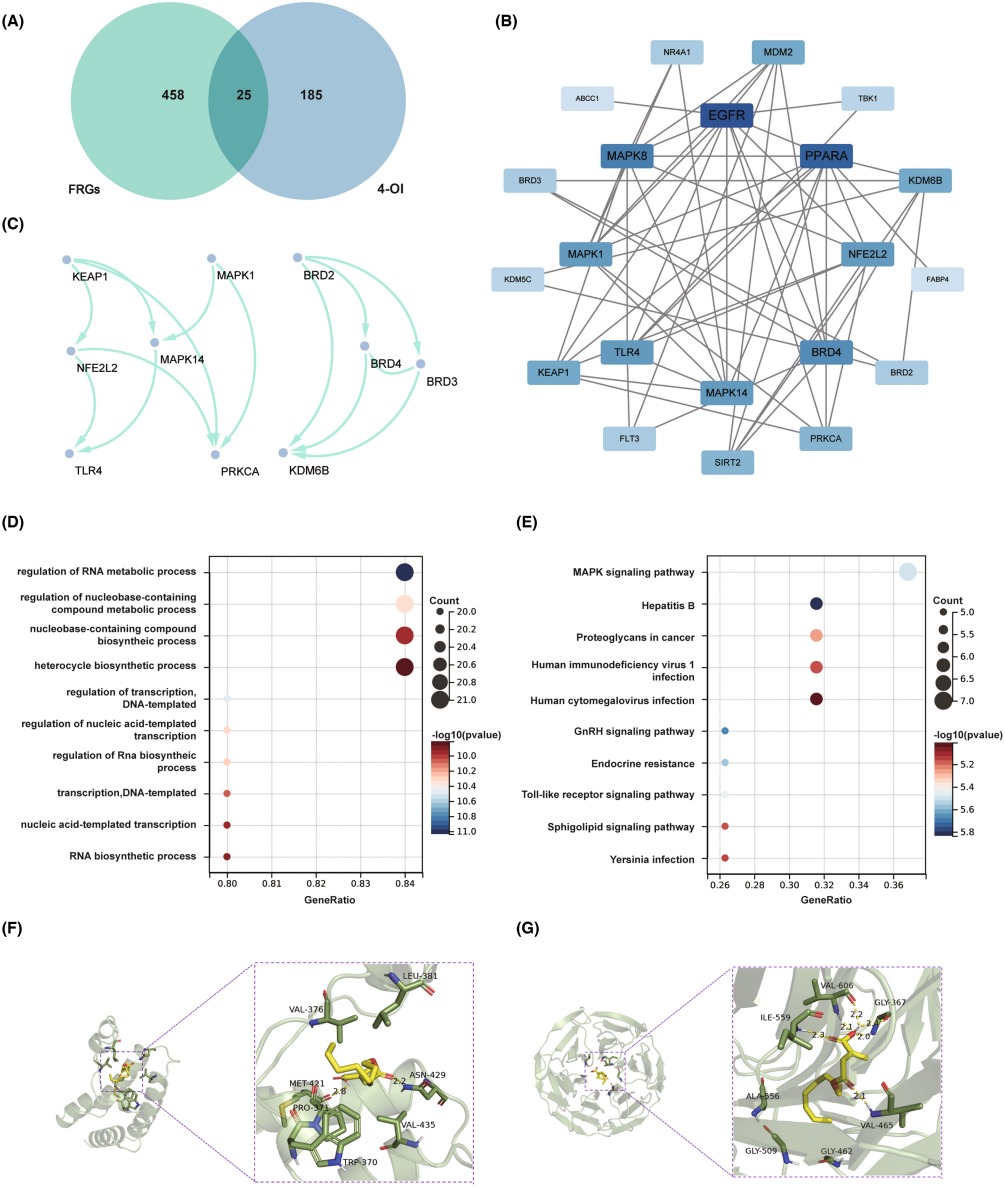
Network pharmacological analysis of the association of 4‐OI with ferroptosis. (A) Venn diagram of 4‐OI targets predicted with ferroptosis driver genes and ferroptosis suppressor genes. FRGs, ferroptosis related genes. (B) Protein–protein interaction (PPI) network of intersecting targets ranked according to degrees. (C) Two subnetworks obtained by the MCODE plugin in Cytoscape. (D) GO terms of biological process based on GO enrichment analysis of the intersecting genes. (E) KEGG enrichment analysis of the intersecting genes. (F) Molecular docking of 4‐OI with BRD2. (G) Molecular docking of 4‐OI with KEAP1.

A network pharmacological analysis was performed to evaluate the potential involvement of 4‐OI in the ferroptosis process (Figure 5; Table S2). A total of 113 predicted targets obtained from the SuperPred database were merged with 106 predicted targets from the SwissTargetPrediction database, resulting in a total of 210 potential targets for 4‐OI after the nine duplicates were removed. A total of 264 ferroptosis driver genes and 238 ferroptosis inhibitory genes obtained from the FerrDb V2 database were considered directly associated with ferroptosis outcome. Venn diagram analysis revealed a total of 25 intersecting targets for 4‐OI (Figure 5A). The PPI network constructed based on degrees revealed that EGFR, PPARA, MAPK8, BRD4, MAPK14, NFE2L2 and TLR4 were ranked prominently (Figure 5B). Two subnetworks were identified using MCODE algorithm. Subnetwork 1 (Score: 2.4) consisted of BRD2, BRD3, BRD4 and KDM6B, while subnetwork 2 (Score: 2.286) contained NFE2L2, TLR4, MAPK1, PRKCA, MAPK14 and KEAP1, with KEAP1 revealed upstream effects in the network (Figure 5C). GO enrichment analysis revealed the biological processes with 4‐OI involved to regulate the ferroptosis outcome. Enriched functions included regulation of RNA metabolic process, regulation of nucleobase‐containing compound metabolic process, nucleobase‐containing compound biosynthetic process and nucleic acid‐templated transcription, suggesting the strong involvement of 4‐OI in nucleic acid metabolism and synthesis (Figure 5D). KEGG pathway enrichment analysis indicated that the MAPK signalling pathway was the most significantly changed pathway with the participation of 4‐OI in the ferroptosis process (Figure 5E). Due to the upstream effects of both BRD2 and KEAP1, molecular docking was performed to investigate the interactions between 4‐OI and these two target proteins (Figure 5F,G). The binding energies of −5.2 kcal/mol and − 5.7 kcal/mol suggested favourable binding interactions between 4‐OI and both proteins, indicating that 4‐OI was probably involved in the ferroptosis process through binding to or modifying BRD2 and/or KEAP1.

**FIGURE 6:**
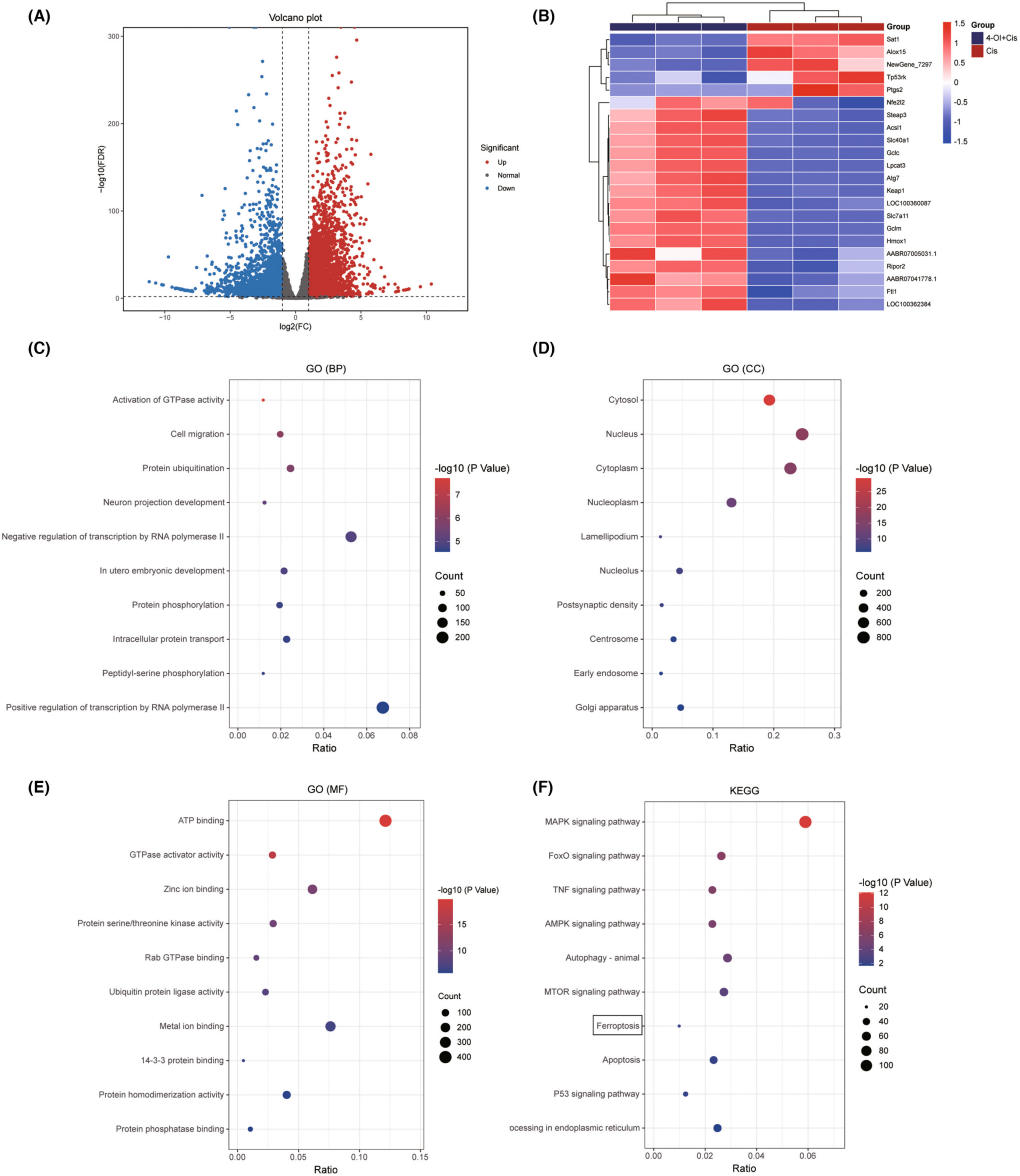
Differentially expressed genes (DEGs) and their KEGG and GO enrichment analyses. (A) Volcano plot of all DEGs presented in blue dots and red dots for down‐ and up‐regulated genes, respectively. (B) Heatmap of clustering of ferroptosis‐related genes presented in red for relatively high expression and blue for relatively low expression. (C–E) GO enrichment analysis based on DEGs with genes annotated in GO terms of (C) biological process (BP), (D) cellular component (CC) and (E) molecular function (MF), respectively. (F) KEGG enrichment analysis based on DEGs.

Transcriptome sequencing and bioinformatics analysis of DEGs were performed to further investigate the protective effect of 4‐OI on cisplatin‐induced ferroptosis process. Based on the RNA‐seq analysis of three groups of cells each of three biological replicates, that is, a total of nine sequencing samples, a total of 5176 significantly differentially expressed genes were identified in the comparison of 4‐OI + cisplatin versus cisplatin groups, with 2431 genes downregulated and 2745 genes upregulated (Figure 6A; Table S3). Subsequently, the GO annotations of these DEGs were performed to identify the GO terms in three categories, that is, biological processes (BP), molecular functions (MF) and cellular components (CC). The top 10 enriched GO terms were presented in bubble pots (Figure 6C–E; Table S4). The ferroptosis pathways were enriched based on the KEGG enrichment analysis (Figure 6F; Table S4). The DEGs related to ferroptosis (Figure 6B) were presented in heatmaps, showing that the intervention of 4‐OI increased the expression of ferroptosis inhibitor genes, such as *Atg7*, *Gclc*, *Gclm*, *Hmox1*, *Slc7a11* and *Ftl1*.

### 3.6 4‐OI inhibits cisplatin‐induced ferroptosis by activating the NRF2/HO‐1 signalling pathway

**FIGURE 7:**
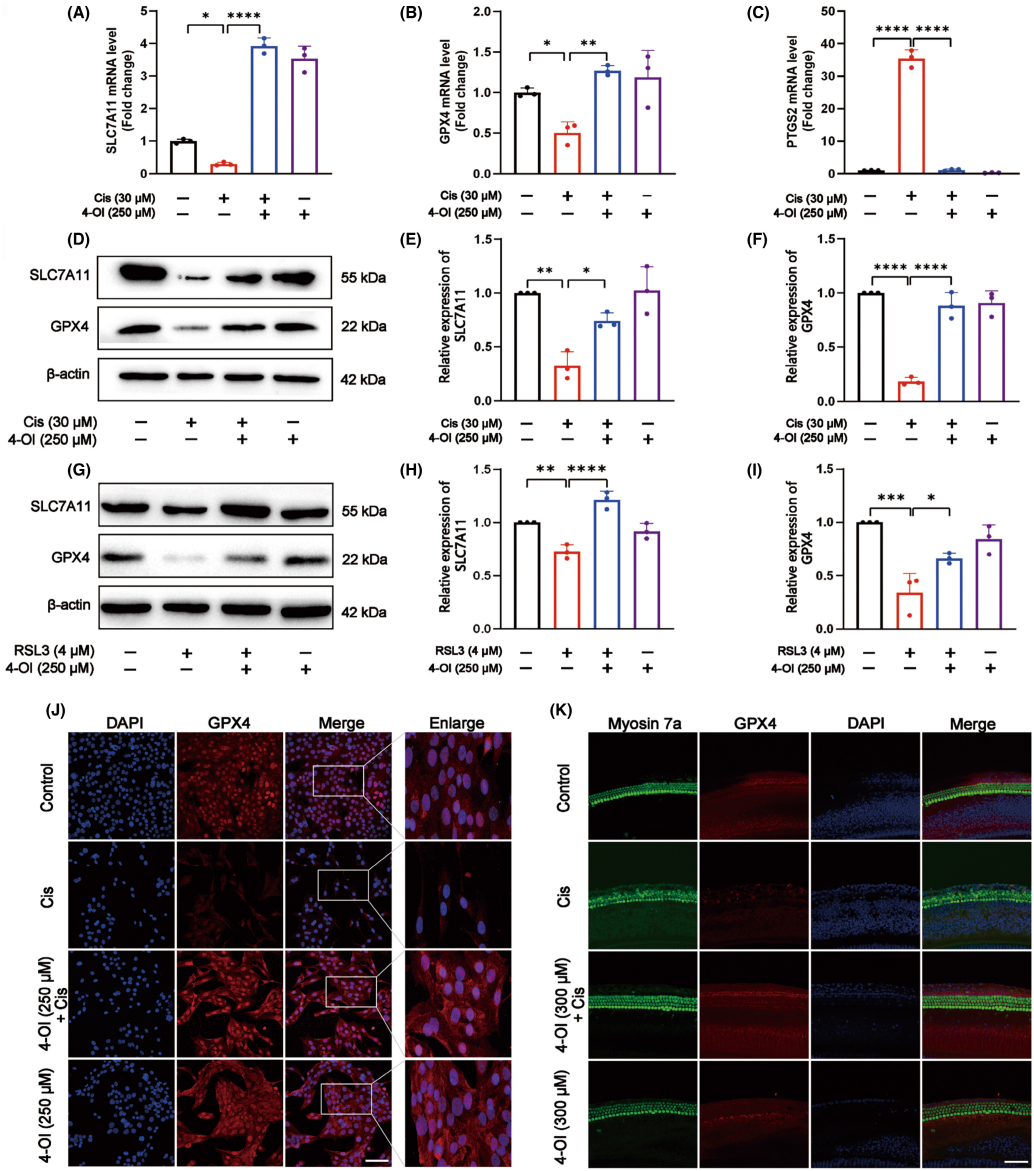
4‐OI inhibits cisplatin‐induced ferroptosis. (A–C) Relative mRNA levels of *SLC7A11*, *GPX4* and *PTGS2* in four groups of HEI‐OC1 cells. (D) Western blot analysis of SLC7A11 and GPX4 in four groups of HEI‐OC1 cells, with cisplatin used for modelling. (E, F) Quantitative statistics of SLC7A11 and GPX4 proteins shown in (D). (G) Western blot analysis of SLC7A11 and GPX4 in four groups of HEI‐OC1 cells, with RSL3 used for modelling. (H, I) Quantitative statistics of proteins SLC7A11 and GPX4 shown in (G). (J) GPX4 immunofluorescence images of four groups of HEI‐OC1 cells (GPX4 and DAPI double staining). (K) Representative images of the cochlear basement membranes of four groups of samples transferred to Myosin 7a (green), GPX4 (red) and DAPI (blue) immunofluorescence staining. The values are expressed as mean ± SEM (*n* = 3). The significant differences are determined by *p < 0.05, **p < 0.01, ***p < 0.001 and ****p < 0.0001, respectively. Scale bar = 40 μm. Cis, cisplatin.

Based on the positive results derived from network pharmacology and RNA‐seq analyses (Figures 5 and 6), the inhibitory effect of 4‐OI on cisplatin‐induced ferroptosis was further evaluated in HEI‐OC1 cells and mice cochlear hair cells. The results obtained from HEI‐OC1 cells indicated that the mRNA levels of both *SLC7A11* and *GPX4* were greatly reduced and the mRNA levels of *PTGS2* were highly increased in the cisplatin group compared with the control, whereas the 4‐OI pretreatment group showed reversed changes in the mRNA levels of these genes (*p* < 0.05; Figure 7A–C). The protein expressions of SLC7A11 and GPX4 were also significantly increased in the 4‐OI pretreatment group compared to the cisplatin group (*p* < 0.05; Figure 7D–F).

In order to further evaluate the protective effect of 4‐OI against ferroptosis, we initially exposed HEI‐OC1 cells to the ferroptosis‐inducing agent RSL3. The CCK‐8 assay revealed that after a 24‐h treatment with 4 μM RSL3, cell viability was significantly dropped to 50% (*p* < 0.05; Figure S1A). To validate whether 4‐OI could alleviate the damage caused by the ferroptosis inducer RSL3, we pretreated HEI‐OC1 cells with 250 μM 4‐OI for 3 h, followed by co‐culturing with RSL3 for an additional 24 h. In comparison to the group treated solely with RSL3, there was a marked increase in cell viability (*p* < 0.05; Figure S1B). These findings strongly indicated that 4‐OI exerted a potent protective effect against RSL3‐induced damage in HEI‐OC1 cells. Furthermore, after modelling with RSL3 on HEI‐OC1 cells, the results of Western blot for SLC7A11 and GPX4 were consistent with those after cisplatin treatment (*p* < 0.05; Figure 7G–I). The immunofluorescence results of HEI‐OC1 cells (Figure 7J) and cochlear hair cells of mice (Figure 7K and Figure S2A,B) showed that the GPX4 fluorescence signal intensity was significantly reduced in the cisplatin group, but significantly increased in the 4‐OI pretreatment group; these results were consistent with those of real‐time PCR and Western blot analyses.

**FIGURE 8:**
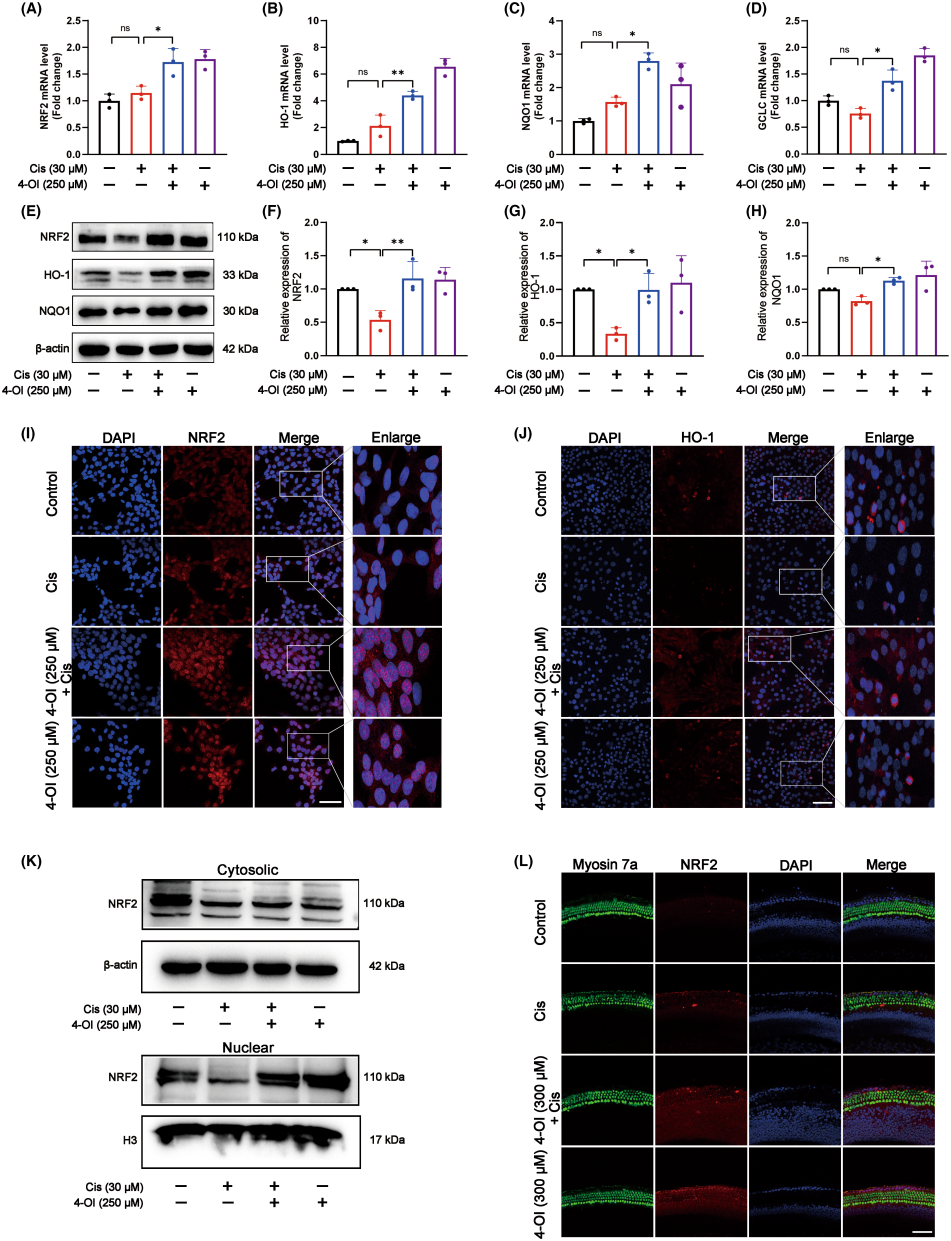
4‐OI alleviates cisplatin‐induced damage to HEI‐OC1 cells and cochlear hair cells by activating NRF2/HO‐1 signalling pathway. (A–D) Relative mRNA levels of *NRF2*, *HO‐1*, *NQO1* and *GCLC* in four groups of HEI‐OC1 cells. (E) Western blot analysis of NRF2, HO‐1 and NQO1 in four groups of HEI‐OC1 cells. (F–H) Quantitative statistics of NRF2, HO‐1 and NQO1 proteins shown in (E). (I, J) Immunofluorescence images of NRF2 and HO‐1 in four groups of HEI‐OC1 cells (double staining of NRF2, HO‐1 and DAPI). (K) Western blot analysis of NRF2 of cytosolic and nuclear origins in four groups of HEI‐OC1 cells. (L) Representative images of immunofluorescence staining of Myosin 7a (green), NRF2 (red) and DAPI (blue) in the middle turn of the cochlear basement membrane in four groups of samples. The values are expressed as mean ± SEM (*n* = 3). The significant differences are determined by *p < 0.05 and **p < 0.01, respectively. ‘ns’ indicates no significance. Scale bar = 40 μm. Cis, cisplatin.

To investigate the preventive effect of 4‐OI against cisplatin ototoxicity, the real‐time PCR was performed to determine the expression of antioxidant element (ARE), an important antioxidant factor associated with NRF2 activation. The results showed that the mRNA expressions of genes encoding *NRF2*, *HO‐1*, NAD(P)H dehydrogenase, quinone 1 (*NQO1*) and glutamate‐cysteine ligase catalytic subunit (*GCLC*), were significantly increased in the 4‐OI pretreatment group compared to the cisplatin group of HEI‐OC1 cells (*p* < 0.05; Figure 8A–D).The expression levels of the of NRF2, HO‐1 and NQO1 were decreased in the cisplatin group compared with the control, whereas the cisplatin‐induced decrease in NRF2 content was reversed by 4‐OI pretreatment and the expressions of the downstream target genes encoding HO‐1 and NQO1 were enhanced (Figure 8E–H). The results of HO‐1 immunofluorescence were consistent with those of the Western blot analysis (Figure 8J). Both the immunofluorescence (Figure 8I) and Western blot (Figure 8K) analysis were used to evaluate the effect of the pretreatment of 4‐OI on the nuclear translocation of NRF2 in cisplatin‐induced HEI‐OCI cells. The results revealed that in the cisplatin group, the expression level of NRF2 in the nucleus was reduced, whereas the nuclear translocation of NRF2 was significantly evident and the expression was increased by the 4‐OI pretreatment. The immunofluorescence staining of NRF2 in the cochlear hair cells showed the consistent patterns with that of the model cells (Figure 8L and Figure S2C,D). These results suggested that the preventive effect of 4‐OI on cisplatin‐induced ferroptosis was probably associated with the activation of the NRF2/HO‐1 signalling pathway.

### 3.7 4‐OI alleviates cisplatin‐induced hearing loss in C57BL/6J mice

**FIGURE 9:**
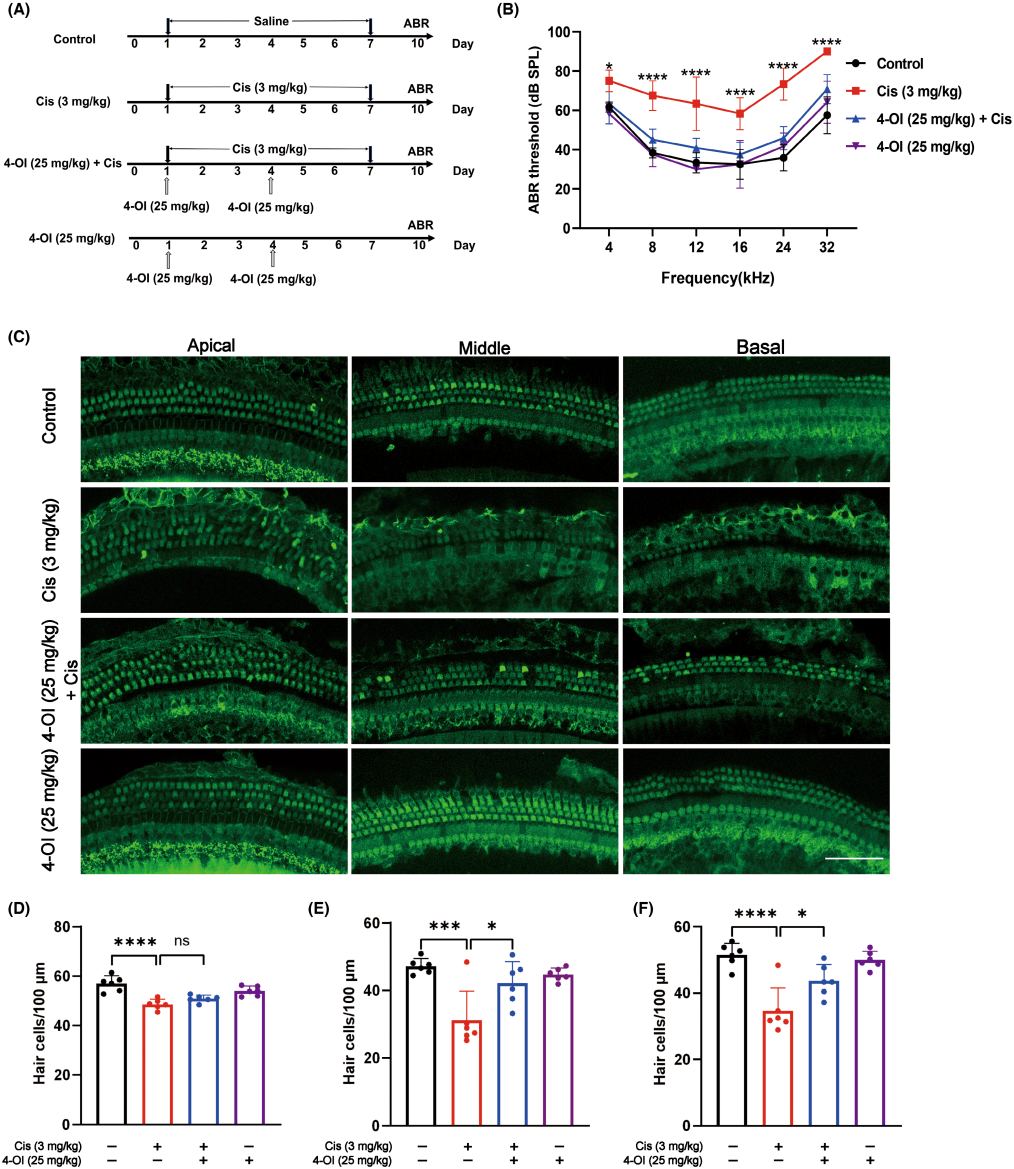
Protective effect of 4‐OI against cisplatin‐induced hearing loss in adult C57BL/6J mice. (A) The specific dosing regimen of 4‐OI and cisplatin was administered via intraperitoneal injection in 8‐week‐old adult male mice. (B) The auditory brainstem response (ABR) thresholds in mice from four experimental groups following pure tone stimulation. (C) Representative images of Myosin 7a (green) in the apical, middle and basal turns of the cochlear basement membrane based on immunofluorescence staining in four groups of samples. (D–F) Number of Myosin 7a positive hair cells per 100 μm at the apical, middle and basal turns of the mice cochlear basement membrane in the four groups of samples shown in (C). The values are expressed as mean ± SEM (*n* = 6). The significant differences are determined by *p < 0.05, ***p < 0.001 and ****p < 0.0001, respectively. ‘ns’ indicates no significance. Scale bar = 40 μm. Cis, cisplatin.

To better evaluate the protective effect of 4‐OI against cisplatin‐induced hearing loss, we used C57BL/6J mice as an in vivo animal experimental model, with the specific design outlined in Figure 9A. The control group of mice displayed consistent daily activity and a slight increase in body weight during the administration period. Conversely, mice treated with cisplatin experienced a decline in body weight and exhibited suboptimal mental states, yet no fatalities were recorded. Notably, the 4‐OI + cisplatin group demonstrated a considerable reduction in these adverse effects compared to the cisplatin group. Moreover, no notable variances were observed between the 4‐OI group and the control group (Figure S3). ABR measurements were employed to assess changes in hearing thresholds before and after treatment in mice. Following 7 consecutive days of intraperitoneal cisplatin administration, there was a significant increase in hearing thresholds at frequencies of 8, 12, 16, 24 and 32 kHz. Pretreatment with 4‐OI before cisplatin significantly reduced the hearing thresholds at all frequencies, with an average reduction of 10 dB at 8, 12 and 16 kHz, and an average reduction of 20 dB at 24 and 32 kHz (*p* < 0.05; Figure 9B). Additionally, results from inner and outer hair cell fluorescence staining and cell counting (*p* < 0.05; Figure 9C–F) demonstrated that, compared to the control group, mice in the cisplatin treatment group exhibited substantial loss of outer hair cells in the middle and basal turns of the cochlear basement membranes, along with disrupted cell arrangement, consistent with the ABR results. After pretreatment with 4‐OI, the loss of middle and basal turn outer hair cells was significantly alleviated, with the structural integrity and orderly arrangement of the cells maintained. In the group treated with 4‐OI alone, no significant differences were observed in hearing thresholds or survival rates of inner and outer hair cells compared to the control group. The results of the in vivo experiments in mice were consistent with our findings in HEI‐OC1 cells and mouse cochlear explants, further demonstrating the protective effect of 4‐OI against cisplatin ototoxicity.

## 4 DISCUSSION

It has been documented that cisplatin primarily impairs auditory function by causing the loss of inner ear hair cells and spiral ganglia^45^, ^46^ and that 4‐OI possesses cell‐permeable properties and exhibits potent protective effects against oxidative stress and inflammation.^33^, ^34^ However, the preventive effect of 4‐OI on cisplatin‐induced ototoxicity has not been investigated to date. In the present study, we examined whether the 4‐OI possesses a protective effect against cisplatin‐induced damage on HEI‐OC1 cells, cochlear explants and adult mice hearing. In this work, we revealed that the loss of inner and outer hair cells became more severe with increasing cisplatin concentration when cisplatin was applied to cochlear explants from 3‐ or 4‐day‐old mammals. Moreover, we found that the basement membrane of the cochlear base turn was more susceptible to cisplatin, suggesting that patients tend to present with bilateral high‐frequency hearing impairment in the pre‐existing stage of hearing damage. The results indicate that pretreatment with 4‐OI significantly reduces the loss of HEI‐OC1 cells, inner hair cells and outer hair cells, underscoring its notable preventive effect against cisplatin‐induced ototoxicity. In the in vivo experiments, the results of ABR audiometry showed that pretreatment with 4‐OI reduced the hearing thresholds of mice in response to stimuli across all frequencies.

In addition to apoptosis, autophagy and necrosis relevant to cisplatin ototoxicity, a growing number of studies suggest that it may also cause hearing loss through other mechanisms.^47^ Our results clearly showed that cisplatin treatment leads to the release of large amounts of ROS and inflammatory factors and that the excessive generation of ROS is a prerequisite for ferroptosis, while the inflammatory responses could disrupt the normal intracellular iron metabolism and regulation.^16^ Therefore, we speculate that cisplatin‐induced cell damage may be caused by activating ferroptosis. To further verify our hypothesis, we analysed the expression of ferroptosis proteins. Our study exhibited that mRNA and protein levels of GPX4 and SLC7A11 gradually declined with prolonged cisplatin treatment of HEI‐OC1 cells, suggesting the occurrence of ferroptosis during cisplatin‐induced ototoxicity. Studies have shown that the inhibition of GPX4 or SLC7A11 impedes the degradation of lipid peroxides, leading to intracellular accumulation of ROS and subsequent ferroptosis.^48^, ^49^, ^50^ Thus, the above evidence suggests that ferroptosis is one of the mechanisms underpinning the cisplatin‐induced ototoxicity.

Prior attempts to explore ferroptosis in the models of cisplatin ototoxicity have utilized the ferroptosis inhibitor ferrostatin‐1 (FER‐1) to attenuate cisplatin‐induced damage in cellular, cochlear explant and animal models.^15^, ^20^ So far, no other drug has been reported to have a protective effect by inhibiting cisplatin induced ferroptosis. Therefore, we investigated whether 4‐OI played a significant cisplatin ototoxic protective effect by inhibiting ferroptosis. Available data have shown that 4‐OI attenuated ferroptosis, which was a key factor in sepsis‐induced acute lung injury.^44^ The results of our RNA‐seq analysis based on the model of cisplatin‐induced ototoxicity implied that 4‐OI acted to inhibit ferroptosis. The subsequent findings in our studies further confirmed that pretreatment with 4‐OI significantly upregulated the mRNA and protein expression levels of both GPX4 and SLC7A11, while reducing the levels of both ROS and inflammatory factors compared to the cisplatin group, indicating that 4‐OI could inhibit cisplatin‐induced ferroptosis.

Next, we further studied the specific mechanism pathway of 4‐OI inhibiting cisplatin induced ferroptosis. In this work, we verified the anti‐ferroptosis effect of 4‐OI through network pharmacological analysis. The results of molecular docking revealed favourable binding activity between 4‐OI and KEAP1. The KEAP1 sequesters NRF2 in the cytoplasm by forming a complex and mediates its degradation via the ubiquitin‐proteasome system.^51^ In the current study, we hypothesized that the binding of 4‐OI to KEAP1 could disrupt the KEAP1 native action and 4‐OI could induce NRF2 nuclear translocation by alkylating KEAP1. In addition, further studies are needed to provide more convincing evidence of 4‐OI binding to KEAP1. Data exhibited that the pretreatment of 4‐OI increased NRF2 expression and nuclear translocation as well as mRNA and protein expressions of NRF2 target genes HO‐1, NQO1, GPX4 and SLC7A11, suggesting that 4‐OI probably inhibited ferroptosis by activating the NRF2/HO‐1 signalling pathway, thereby preventing cisplatin ototoxicity. Therefore, pharmacologically regulating NRF2 signalling pathway, targeting upstream regulators of ferroptosis, such as ROS production and ferroptosis‐related proteins GPX4 and SLC7A11, is an ideal approach to treat cisplatin ototoxicity.

Generally, with the extension of treatment time and increased dosage, most drugs tend to show adverse reactions, greatly limiting their clinical applications. Our study based on in vivo and in vitro experiments revealed no significant cytotoxicity at certain doses of 4‐OI, suggesting the high potential of 4‐OI as a possible drug used to prevent cisplatin ototoxicity. For the first time, our study has shown that 4‐OI acted as a novel NRF2 activator to effectively attenuate the cellular damage caused by cisplatin, suggesting the significant promise of 4‐OI as a therapeutic intervention to attenuate cisplatin ototoxicity in the clinical setting.

In conclusion, 4‐OI effectively mitigated cisplatin‐induced hearing loss in mice and preserved HEI‐OC1 cell and cochlear hair cell viability, possibly through the activation of the NRF2/HO‐1 signalling pathway. Encouragingly, 4‐OI holds promise as a novel drug for the prevention of cisplatin‐associated hearing impairment. Further research is warranted to elucidate the precise molecular mechanisms underlying the protective effects of 4‐OI and to investigate the interplay among relevant metabolic pathways using with inhibitor blocking or target gene knockout in animal in vivo experiments.

## AUTHOR CONTRIBUTIONS

**Li Zhang:** Conceptualization (equal); formal analysis (equal); investigation (equal); methodology (equal); writing – original draft (equal). **Wenao Song:** Conceptualization (equal); investigation (equal); methodology (equal). **Hua Li:** Investigation (equal); methodology (equal). **Xiaolin Cui:** Conceptualization (equal); methodology (equal); writing – original draft (equal). **Jingyu Ma:** Investigation (equal); methodology (equal). **Rongrong Wang:** Methodology (equal). **Yue Xu:** Formal analysis (equal). **Ming Li:** Formal analysis (equal). **Xiaohui Bai:** Conceptualization (equal); methodology (equal); writing – review and editing (equal). **Dawei Wang:** Project administration (equal); supervision (equal). **Haihui Sun:** Project administration (equal); supervision (equal). **Zhiming Lu:** Project administration (equal); supervision (equal).

## CONFLICT OF INTEREST STATEMENT

The authors declare that they have no conflict of interest.

## Supporting information


Figures S1–S3.

Table S1.

Table S2.

Table S3.

Table S4.

## Data Availability

The data used to support the findings of this study are available from the corresponding author upon reasonable request.
