# Correction to “4‐Octyl Itaconate Alleviates Cisplatin‐Induced Ferroptosis Possibly via Activating the NRF2/HO‐1 Signaling Pathway”

**DOI:** 10.1111/jcmm.71086

**Published:** 2026-03-10

L. Zhang, W. Song, H. Li, et al., “4‐octyl Itaconate Alleviates Cisplatin‐Induced Ferroptosis Possibly via Activating the NRF2/HO‐1 Signalling Pathway,” *Journal of Cellular and Molecular Medicine* 28, no. 7 (2024): e18207. https://doi.org/10.1111/jcmm.18207

In Figure S2C, the DAPI image of the cochlear Apical in the cisplatin‐treated group does not correspond to the DAPI channel (blue) in the final merged image. This error occurred due to a mistake during image layout arrangement. The correct layout is shown below. We would like to emphasize that this error does not affect any of the study's findings or final conclusions, and it allows for any reproducibility experiments to confirm the results.

**FIGURE S2:**
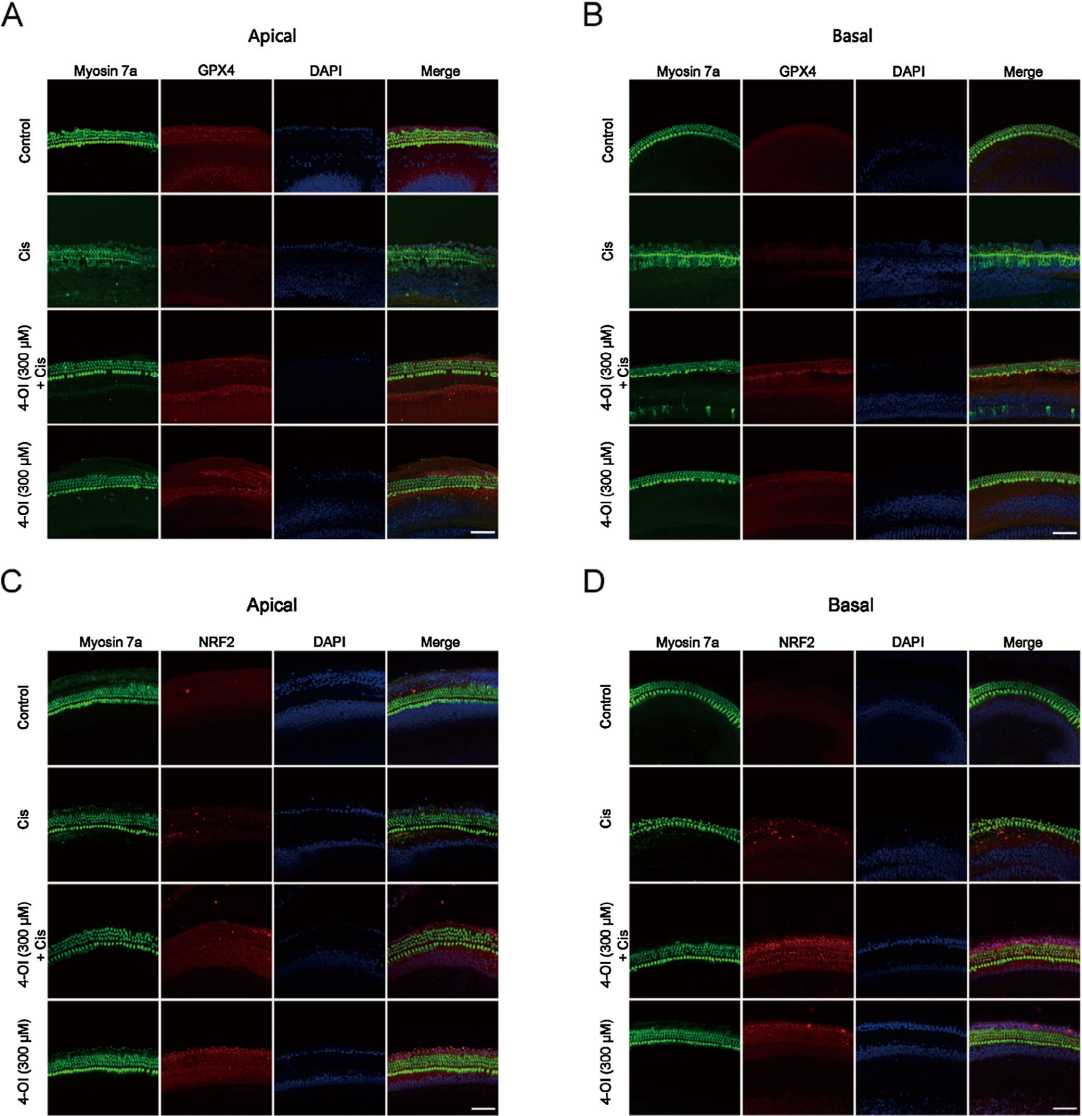
4‐OI inhibits cisplatin‐induced ferroptosis. (A, B) Representative images of immunofluorescence staining of Myosin 7a (green), GPX4 (red), and DAPI (blue) in the apical and basal turns of the cochlear basement membrane in four groups of samples. (C, D) Representative images of immunofluorescence staining of Myosin 7a (green), NRF2 (red), and DAPI (blue) in the apical and basal turns of the cochlear basement membrane in four groups of samples. Scale bar = 40 μm. Cis, cisplatin.

We sincerely apologize for any inconvenience caused to the readers and the publisher.

