# Supplementary material for: 4‐octyl itaconate alleviates cisplatin‐induced ferroptosis possibly via activating the NRF2/HO‐1 signalling pathway
Source: J Cell Mol Med. 2024 Mar 20;28(7):e18207. doi: 10.1111/jcmm.18207 (PMC10951885; doi:10.1111/jcmm.18207)
Supplement: Supplementary file 1 — Figures S1–S3. [file JCMM-28-e18207-s002.docx]

**Supplementary Material**

4-octyl itaconate alleviates cisplatin-induced ferroptosis possibly via activating the NRF2/HO-1 signaling pathway

Li Zhang^1^, Wenao Song^2^, Hua Li^3^, Xiaolin Cui^2^, Jingyu Ma^1^, Rongrong Wang^2^, Yue Xu^2^, Ming Li^3^, Xiaohui Bai^1,2^, Dawei Wang^4^, Haihui Sun^5^& Zhiming Lu^1,2^

Correspondence should be addressed to Zhiming Lu ([luzhiming@sdu.edu.cn](mailto:luzhiming@sdu.edu.cn)) and Haihui Sun ([sunhaihui569@sina.com](mailto:sunhaihui569@sina.com))

**
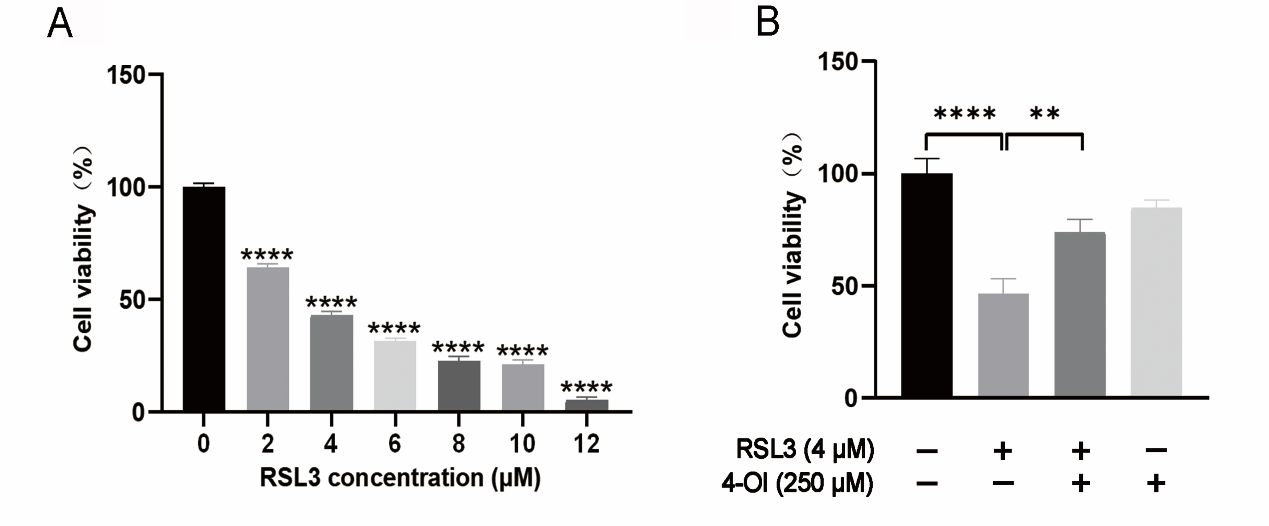
**

**FIGURE S1** 4-OI alleviates the RSL3-induced decrease in HEI-OC1 cell viability. (A) Cell survival rate by CCK8 assay after 24 h treatment of different concentrations of RSL3 (0, 2, 4, 6, 8, 10, and 12 μM) on HEI-OC1 cells. (B) Cell survival rate based on CCK8 of HEI-OC1 cells first treated with 250 μM 4-OI for 3 h and then by 4 μM RSL3 for 24 h. The values are expressed as mean ± SEM (n = 3). The significant differences are determined by *P* < 0.01 (**) and *P* < 0.0001 (****), respectively.


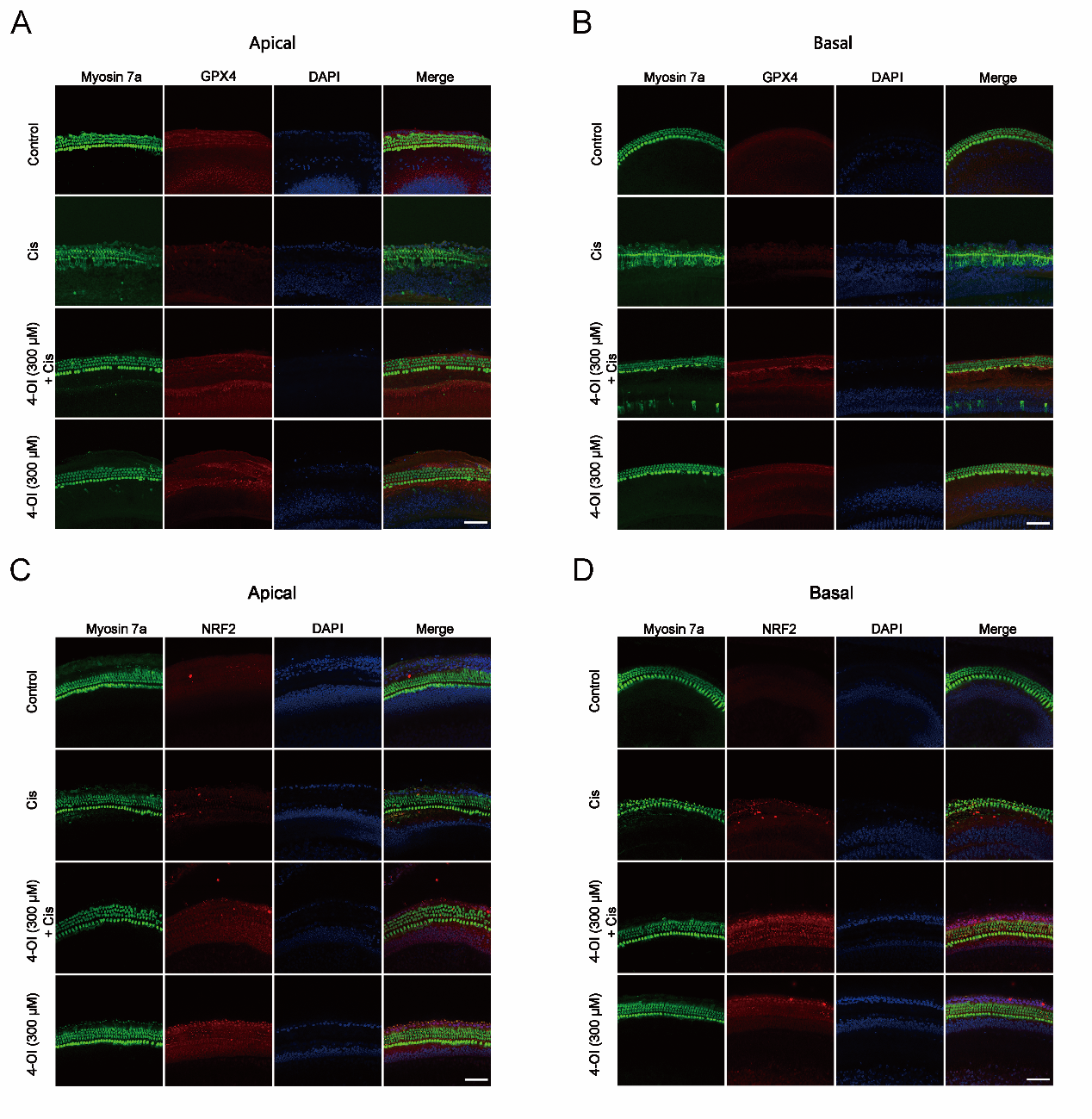
 **FIGURE S2** 4-OI inhibits cisplatin-induced ferroptosis. (A, B) Representative images of immunofluorescence staining of Myosin 7a (green), GPX4 (red), and DAPI (blue) in the apical and basal turns of the cochlear basement membrane in four groups of samples. (C, D) Representative images of immunofluorescence staining of Myosin 7a (green), NRF2 (red), and DAPI (blue) in the apical and basal turns of the cochlear basement membrane in four groups of samples. Scale bar = 40 μm. Cis, cisplatin.


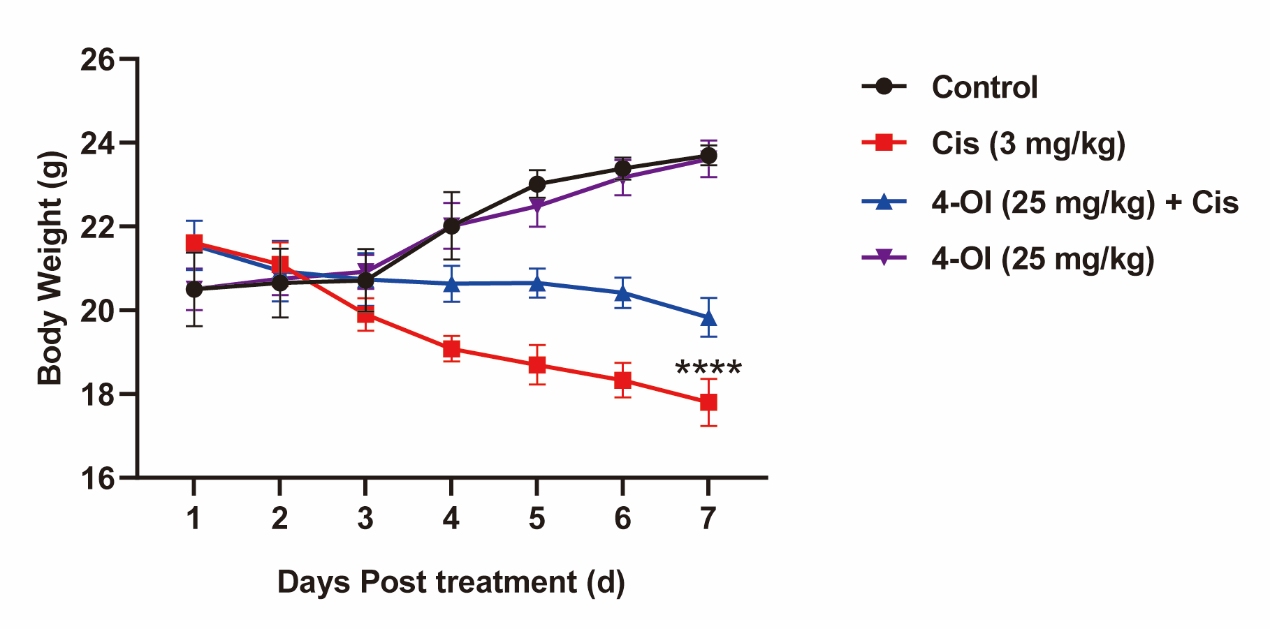


**FIGURE S3** Weight change curve of four groups of experimental mice. The values are expressed as mean ± SEM (n = 6). The significant differences are determined by *P* < 0.0001 (****). Cis, cisplatin.
