# Supplementary material for: 4‐octyl itaconate alleviates cisplatin‐induced ferroptosis possibly via activating the NRF2/HO‐1 signalling pathway
Source: J Cell Mol Med. 2024 Mar 20;28(7):e18207. doi: 10.1111/jcmm.18207 (PMC10951885; doi:10.1111/jcmm.18207)
Supplement: Supplementary file 2 — Table S1. [file JCMM-28-e18207-s001.docx]

**Supplementary Material**

4-octyl itaconate alleviates cisplatin-induced ferroptosis possibly via activating the NRF2/HO-1 signaling pathway

Li Zhang^1^, Wenao Song^2^, Hua Li^3^, Xiaolin Cui^2^, Jingyu Ma^1^, Rongrong Wang^2^, Yue Xu^2^, Ming Li^3^, Xiaohui Bai^1,2^, Dawei Wang^4^, Haihui Sun^5^& Zhiming Lu^1,2^

Correspondence should be addressed to Zhiming Lu ([luzhiming@sdu.edu.cn](mailto:luzhiming@sdu.edu.cn)) and Haihui Sun ([sunhaihui569@sina.com](mailto:sunhaihui569@sina.com))

**Supplementary Table 1**

Primers and their sequences for real-time PCR analysis

| **Name** | **Sequence (5’-3’)** |
| --- | --- |
| *Nrf2* | F: TAGATGACCATGAGTCGCTTGC |
|  | R: GCCAAACTTGCTCCATGTCC |
| *SLC7A11* | F: CCCTGGCATTTGGACGCTAC |
|  | R: CTCCAGCTGACACTCGTGCTATTTA |
| *GPX4* | F: CCCGATATGCTGAGTGTGGTTTA |
|  | R: TTCTTGATTACTTCCTGGCTCCTG |
| *PTGS2* | F: CTGGAACATGGACTCACTCAGTTTG |
|  | R: AGGCCTTTGCCACTGCTTGTA |
| *TNF-α* | F: ACTCCAGGCGGTGCCTATGT |
|  | R: CTGACGAAGTGACGCCATCTG |
| *IL-1β* | F: TCCAGGATGAGGACATGAGCAC |
|  | R: GAACGTCACACACCAGCAGGTTA |
| *IL-6* | F: CCACTTCACAAGTCGGAGGCTTA |
|  | R: TGCAAGTGCATCATCGTTGTTC |
| *HO-1* | F: CTGGAGATGACACCTGAGGTCAA |
|  | R: CTGACGAAGTGACGCCATCTG |
| *NQO1* | F: TGGCCGAACACAAGAAGCTG |
|  | R: GCTACGAGCACTCTCTCAAACC |
| *GCLC* | F: GCACATCTACCACGCAGTCAA |
|  | R: ACATCGCCTCCATTCAGTAACAAC |
